# The role of MDM2–p53 axis dysfunction in the hepatocellular carcinoma transformation

**DOI:** 10.1038/s41420-020-0287-y

**Published:** 2020-06-19

**Authors:** Hui Cao, Xiaosong Chen, Zhijun Wang, Lei Wang, Qiang Xia, Wei Zhang

**Affiliations:** 1 Department of Liver Diseases, Longhua Hospital, Shanghai University of Traditional Chinese Medicine, Shanghai, 200030 China; 2 Department of Liver Surgery, Renji Hospital, School of Medicine, Shanghai Jiaotong University, Shanghai, 200127 China; 3 Department of Traditional Chinese Medicine, Putuo People’s Hospital Affiliated to Tongji University, Shanghai, China

**Keywords:** Chemotherapy, Gastrointestinal cancer

## Abstract

Liver cancer is the second most frequent cause of cancer-related death globally. The main histological subtype is hepatocellular carcinoma (HCC), which is derived from hepatocytes. According to the epidemiologic studies, the most important risk factors of HCC are chronic viral infections (HBV, HCV, and HIV) and metabolic disease (metabolic syndrome). Interestingly, these carcinogenic factors that contributed to HCC are associated with MDM2–p53 axis dysfunction, which presented with inactivation of p53 and overactivation of MDM2 (a transcriptional target and negative regulator of p53). Mechanically, the homeostasis of MDM2–p53 feedback loop plays an important role in controlling the initiation and progression of HCC, which has been found to be dysregulated in HCC tissues. To maintain long-term survival in hepatocytes, hepatitis viruses have lots of ways to destroy the defense strategies of hepatocytes by inducing *TP53* mutation and silencing, promoting *MDM2* overexpression, accelerating p53 degradation, and stabilizing MDM2. As a result, genetic instability, chronic ER stress, oxidative stress, energy metabolism switch, and abnormalities in antitumor genes can be induced, all of which might promote hepatocytes’ transformation into hepatoma cells. In addition, abnormal proliferative hepatocytes and precancerous cells cannot be killed, because of hepatitis viruses-mediated exhaustion of Kupffer cells and hepatic stellate cells (HSCs) and CD4^+^T cells by disrupting their MDM2–p53 axis. Moreover, inefficiency of hepatic immune response can be further aggravated when hepatitis viruses co-infected with HIV. Unlike with chronic viral infections, MDM2–p53 axis might play a dual role in glucolipid metabolism of hepatocytes, which presented with enhancing glucolipid catabolism, but promoting hepatocyte injury at the early and late stages of glucolipid metabolism disorder. Oxidative stress, fatty degeneration, and abnormal cell growth can be detected in hepatocytes that were suffering from glucolipid metabolism disorder, and all of which could contribute to HCC initiation. In this review, we focus on the current studies of the MDM2–p53 axis in HCC, and specifically discuss the impact of MDM2–p53 axis dysfunction by viral infection and metabolic disease in the transformation of normal hepatocytes into hepatoma cells. We also discuss the therapeutic avenues and potential targets that are being developed to normalize the MDM2–p53 axis in HCC.

## Introduction

Liver cancer is the second most frequent cause of cancer-related death globally with 854,000 new cases and 810,000 deaths per year, and accounting for 7% of all cancers^1^. The main histological subtype of liver cancer is hepatocellular carcinoma (HCC), which is mainly derived from hepatocytes^2^. According to the epidemiologic studies, the incidence of HCC is increasing progressively with advancing age in all populations and reaching a peak at 70 years; HCC has a strong male preponderance, with a male-to-female ratio estimated to be 2–2.5:1^3^. Approximately 90% of HCCs are associated with a known underlying etiology, including chronic hepatitis virus infections (HBV and HCV), HIV infection, alcohol intake, aflatoxin exposure, metabolic diseases, and cigarette smoking^4^. The mechanisms of the carcinogen-mediated HCC are multipathways of which MDM2–p53 axis dysfunction had been impressive^5^.

Mechanically, the homeostasis of MDM2–p53 axis can be presented with the normal functions of p53 and MDM2, both of which were found abnormally expressed in HCC tissues^6^. Since its discovery 35 years ago, p53 has emerged as a key antitumor factor^7^. The p53 response can be activated by multiple stress signals, such as genotoxic stress, oncogene activation, ribosomal stress, hypoxia, and nutrient fluctuation^8^. After p53 activation, it would activate or repress the expression of numerous genes that relate with cancer initiation/development^9^. Such proapoptotic BCL‑2 family proteins [BCL‑2‑associated X protein (BAX), NOXA, and p53-upregulated modulator of apoptosis (PUMA)] can be transcriptionally activated^10^; metabolic enzymes [p53-induced glycolysis and apoptosis regulator (TIGAR) and glucose transporters] that contributed to cancer metabolic switch can be inhibited^11^. Indeed, accumulating evidence suggests that p53 is critical to suppress cancer development^12^. As previously noted, p53 plays multiple roles in cell-cycle arrest, senescence, and apoptosis, all of which might account for the protection of the genome from accumulating and passing these mutations to the daughter cells^13^. Hence, p53 has been considered as the “guardian of the genome”^14^. The level of cellular p53 protein is subjected to a wide range of post-translational modifications by which its subcellular localization, stability, and conformation can be regulated^15,16^. Under conditions of cellular homeostasis, the stability and function of p53 protein are controlled by MDM2 that targets p53 for degradation and directly inhibits p53 activity by binding to the transcriptional activation domain^17^. As an important ubiquitin ligase (E3 ligases), MDM2 combined with its binding partner MDMX to keep p53 activity after p53 transcriptional activities, as well as set up an efficient feedback loop to limit the p53 response^18^. As discussed above, the main risk factors of HCC are chronic viral infections (HBV, HCV, and HIV) and metabolic disease, all of which likely induce HCC initiation by interfering with MDM2–p53 axis dysfunction^19^. Hence, in this review, we focused on the dysfunction of MDM2 and p53 in HCC, and try to propose the possible mechanisms that carcinogens mediated the transformation of normal hepatocytes into hepatoma cells.

## The physiological functions of p53 and MDM2 in normal hepatocytes

Although best known for its activity as a tumor suppressor, p53 is also involved in hepatocyte proliferation, apoptosis, and metabolism^20,21^. After partial hepatectomy, the upregulation of p53 expression can regulate CDK2- and CDK4 activities that contribute to DNA synthesis of hepatocytes, which ultimately promotes liver regeneration^22^. Moreover, if p53^−/−^mice suffer from acute liver failure, the liver injury of mice will be prolonged, and the initiation of liver regeneration will be delayed than that in wild type^23^. However, p53 was found to be accounted for toxicant-induced hepatitis, such as alcohol ingestion, lipid droplet, bile acids, and heavy metal cadmium^24^. Hence, to avoid the overactive hepatocyte apoptosis by intrahepatic inflammation, MDM2 precisely regulated p53 functions that play the protective roles in liver detoxication responses^25^. Because it has an autoregulatory negative feedback loop between p53 and MDM2, toxicant-induced p53 overactivation might enhance MDM2 expression, and thereby promote Akt phosphorylation and inhibit Pim phosphorylation in hepatocytes, which contributed hepatocytes to survival in toxicant-induced hepatic inflammation^26^. Mice would cause severe damage of liver parenchyma after MDM2 deletion^27^. Indeed, hepatocyte apoptosis and hepatic inflammation in p53-dependent manner can be abrogated by some hepatic protectants because they can increase MDM2 expression^28^. Hence, the above results seem to indicate that normal MDM2–p53 aixs is required for cell proliferation and renewal of normal hepatocytes. Likewise, the homeostasis of MDM2–p53 axis is essential for hepatocyte metabolism^29^. To guarantee the energy production of hepatocytes from OXPHOS, p53 exerts its pleiotropic role in limiting glycolysis and diverting pyruvate toward the tricarboxylic acid cycle (TCA)^30^. p53 can regulate the function of multiple molecules that are required for glycolysis by suppressing the glucose transporter type 1 (GLUT1) and GLUT4 transcription^31^, regulating the TIGAR expression, and inhibiting the several glycolytic enzymes’ expression via activation of microRNA‑34a^32^. In addition, the mitochondrial protein glutaminase 2 (GLS2) is the target of p53, which supported mitochondrial respiration and ATP production^33^. Although MDM2 always presents the opposite roles for p53, it might play the same role in glycol metabolism. *MDM*2KO mice exhibited a marked impairment in glucose tolerance on glucose challenge^34^. The glucose deprivation, MDM2, can be upregulated by mTOR via p53-dependent manner, which could promote cellular response to these environmental alterations^35^. However, it is worth noting that the p53 hyperactivation can induce hepatocyte apoptosis classically through the mitochondrial pathway; in the cytoplasts, p53 can lead to a marked disruption of the mitochondrial structure by promoting the Bax/Bak oligomerization, antagonizing Bcl-XL/Bcl-2 antiapoptotic effects, and forming a complex with cyclophilin D^36^. However, as described above, the activity of p53 is tightly controlled by its negative regulator (MDM2); induction of MDM2 expression by p53 will result in returning p53 to a basal level^37^. Hence, the normal liver is relatively resistant to p53-mediated cell death, and the link between mitochondria-mediated apoptosis and p53 activation following DNA damage is rarely observed^38^. Taken together, the homeostasis of MDM2–p53 axis can promote hepatocyte proliferation, xenobiotic detoxification, and OXPHOS-mediated energy metabolism. In addition, MDM2–p53 axis is critical for sensing nutrient deprivation and maintaining liver lipid homeostasis^39^. Hence, perturbations in their balance not only contribute to metabolic disorders but also to cancer development.

## Chronic viral infections promote hepatoma cells’ transformation by inducing MDM2–p53 axis dysfunction

**Fig. 1:**
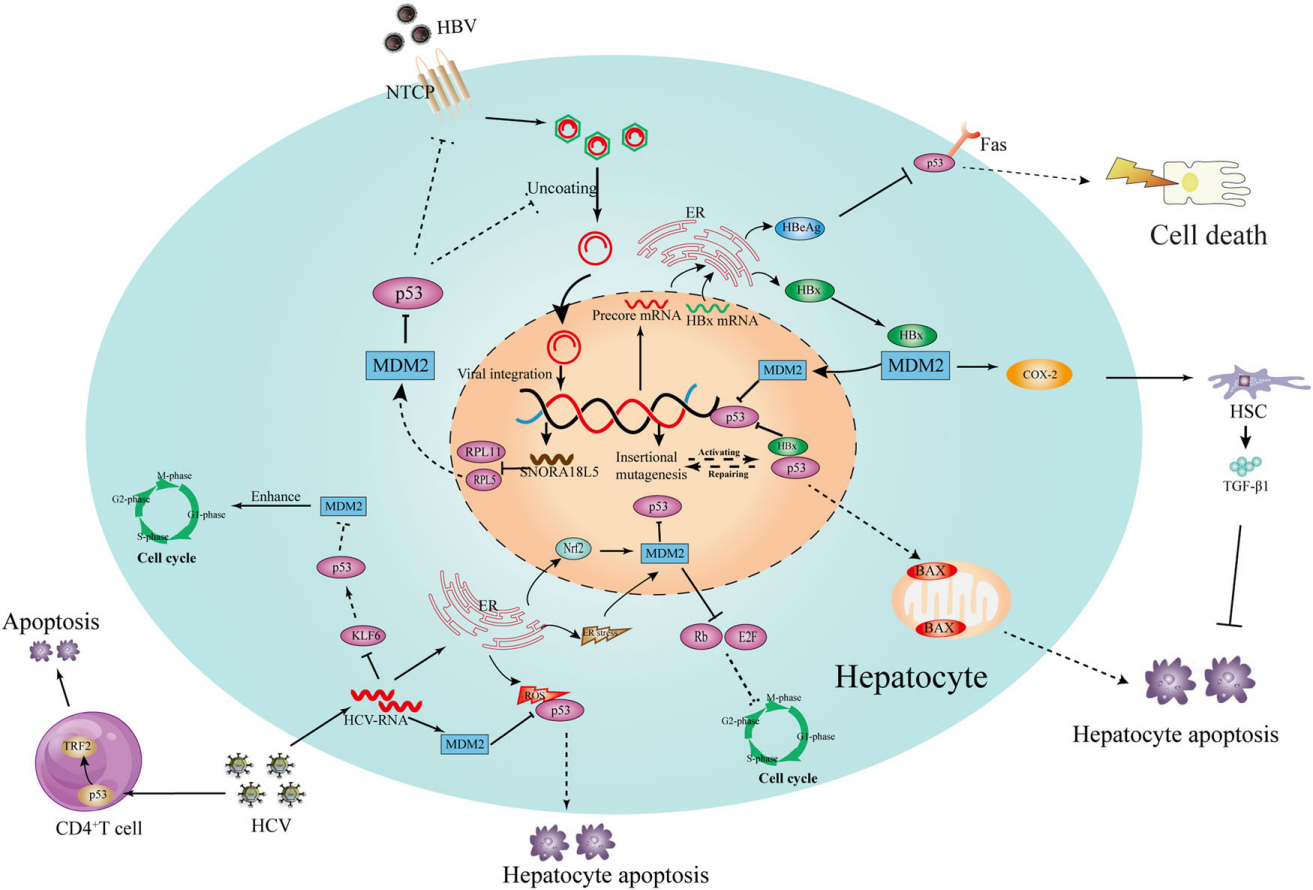
Chronic viral infections mediated abnormal proliferation and apoptosis of hepatocytes by interfering with MDM2–p53 axis. After HBV enters into the cytoplasm via NTCP, the cccDNA of HBV can integrate into host chromosomes, which provoked DNA damage of the host and p53 activation. Normally, two types of pathways can be activated, both of which are related with cell survival inhibitor: p53–Bax–mitochondria-induced apoptosis, and p53/fas-mediated cell death. However, HBx can directly bind to p53 that inhibit the infected hepatocyte apoptosis from p53–Bax–mitochondria. Moreover, HBx can disturb the transcriptional activity of p53 by binding with MDM2, which might impair p53-mediated DNA repairing. In addition, HBx binding to MDM2 can upregulate the expression of COX2 that can promote TGF-β1 expression from HSC, and thereby block hepatocyte apoptosis. Likewise, HBeAg can inhibit the p53/Fas pathway-mediated cell death. In addition, HBV can induce a small nucleolar RNA expression (SNORA18L5) in hepatocytes, which can mediate p53 ubiquitination and degradation by preventing RPL5 and RPL11 escape into the nucleoplasm to bind MDM2. Unlike HBV, the replication of HCV is mainly processed around the ER, which can induce chronic ER stress, oxidative stress, and Nrf2 activation. Nrf2 can induce MDM2-mediated Rb and p53 proteosomal degradation that might block the Rb/E2F pathway-mediated inhibitor of cell-cycle progression of infected hepatocytes. Moreover, ER stress can further promote the cell cycle by increasing MDM2 expression. Likewise, HCV can directly inhibit the expression of KLF6 that can contribute to cell-cycle arrest by promoting p53-induced MDM2 degradation. In addition, HCV can overcome the ROS/p53-mediated apoptosis by promoting MDM2 accumulation and inducing ub-mediated proteasomal degradation of p53. Interestingly, HCV can induce naive CD4^+^T-cell exhaustion via p53-dependent manner, which can avoid infected hepatocytes to be killed by T cells.

To promote themselves long-term survival in the host, viruses have lots of ways to interfere with the defense mechanisms of hepatocytes and immunity system, such as upregulating virus-receptor expression, changing hepatocytes’ metabolic pathways, and inducing hepatic T-cell exhaustion^40^. Among them, viruses-mediated MDM2–p53 axis dysfunction not only involved in their replication, but also induced malignant transformation of hepatocytes (Fig. 1).

## HBV-induced hepatoma cell transformation

In the highest-incidence HCC areas, HBV infection is involved in more than 80% of the total HCC^41^. HBV is a small enveloped DNA virus that covalently closed circular DNA (cccDNA) carries the full genetic information that is required for its replication; cccDNA can integrate into host chromosomes, which was associated with establishment of persistent HBV infection and HCC initiation^42^. Accumulating evidence suggests that there are lots of mechanisms that involved in the transformation of normal hepatocytes into hepatoma cells during long-term HBV infection. Such host cancer genes (*TERT*, *MLL4*, *CCNE1*, *SENP5*, and *ROCK1*), all of which had been found to be upregulated in HCC tissues, can be integrated with cccDNA and thereby induced a classic retrovirus-like insertional mutagenesis that contributed to chromosomal instability of hepatocytes^43^. In addition, multiple viral proteins (HBx, HBc, HBe, and preS) can interfere with hepatocyte functions, activate oncogenic pathways, and sensitize hepatocytes to mutagens^44^.

Mechanically, p53 can directly suppress HBV replication by binding and repressing the HBV enhancer^45^. Recently, Ying Yan et al.^42^ proposed a new mechanism that p53 controls HBV infection; they found that p53 can inhibit the entry of HBV into hepatocytes by binding to sodium taurocholate cotransporting polypeptide (NTCP) promoter region (which is the key receptor to recognize HBV hepatocytes) and thereby inhibiting NTCP synthesis. However, numerous studies have shown that a high frequency of p53 inactivation and upregulation of NTCP expression was seen in chronic HBV infection, both of which are correlated with shorter survival times of patients with HBV-related HCC^46^. Hence, it seems that there are two possible mechanisms that HBV promotes hepatocarcinogenesis without cirrhosis with exhaustion of p53 function (p53 gene mutant/p53 protein inactivation). First, the hepatocytes infected with HBV cannot be killed via p53-dependent pathway, which might contribute to disruption of proliferation and apoptosis of hepatocytes, and favor the accumulation of genetic alterations and even pass those mutations to the daughter cells^47^. Second, the upregulation of NTCP expression would provide more channels by which HBV can easily enter into the nascent proliferating hepatocytes, which might prolong the natural history of HBV infection and contribute to the persistent low-/moderate-grade inflammation in the liver^48^. As Sia D et al. proposed^49^, genetic instability (alterations/mutations) combined with chronic inflammation has greater ability to induce HCC initiation. In general, chronic inflammation-induced liver regeneration could induce proliferation of hepatocytes that were marked by telomerase expression, and were found to be sparsely distributed across different layers of hepatocytes within the lobule^50^. However, one subset of those hepatocytes, which is marked by leucine-rich repeat-containing G-protein-coupled receptor 5 (LGR-5), was found to be involved in the HCC development^51^. Indeed, LGR-5 expression was found to be upregulated in HBV-related HCC tissues, and patients with higher expression of LGR-5 might have a poor outcome^52^. A very recent study seems to find out a link between LGR-5 and HCC initiation: LGR-5 can induce p53 degradation and disrupt its stabilization by interfering with programmed cell death protein 5 (PDCD5) nuclear translocation, which could contribute to the long life of abnormal proliferative hepatocytes^53^. Hence, it seems that LGR-5 telomerase-positive hepatocytes are the main source of inflammatory-induced liver regeneration after HBV long-term infection, and also contribute to abnormal proliferative hepatocytes because HBV–LGR-5 mediated p53 dysfunction. Of note, these mechanisms might directly induce HCC development without cirrhosis. In addition, Pengbo Cao et al.^54^ also found a possible mechanism of HBV-related HCC development without cirrhosis because of MDM2–p53 axis dysfunctions; the expression of small nucleolar RNAH/ACA box 18-like 5 (SNORA18L5) in hepatocytes can be upregulated by HBV, which promotes p53 ubiquitination and degradation by preventing RPL5 and RPL11 (ribosomal proteins) to escape into the nucleoplasm to bind MDM2.

In this context, it seems clearer that multiple HBV proteins can exert their oncogenic roles by interfering with MDM2–p53 axis homeostasis in hepatocytes. HBV X protein (HBx), the X region that encodes polypeptides of 154 amino acids, can not only promote HBV survival in hepatocytes, but also interfere with the normal rhythms of hepatocytes by disturbing the MDM2–p53 axis. Wang XW^55^ proposed that HBx protein can modify p53 conformation by developing HBx–p53 complex, and thereby inhibiting p53 to bind to its DNA consensus sequence, which was similar to p53 missense mutants in human cancers; hence, they considered that it might be a possible mechanism that hepatocytes would differentiate into hepatoma cells, because the p53-dependent damaged DNA repairing pathway was blocked, and the functions of key tumor-suppressor genes of hepatocytes were abolished^56^. A similar study from Ranxu Zhu et al.^57^ found that HBx proteins can induce GAS2 transcriptionally silenced in human hepatocytes, which can inhibit p53-dependent apoptosis and senescence to offset HBx protein-mediated oncogenesis. In addition, p53-mediated activation of microRNA-148a was found to be suppressed by HBx protein, which could promote HCC growth and metastasis because microRNA-148a is a regulator of the HPIP/mTOR pathway activation that is required for virus-related tumor growth and metastasis^58^. Hence, the above evidence seems to suggest that HBx has a stronger ability to impair p53 functions, and is a key protein in HBV-related HCC development and initiation. Indeed, HBx protein-mediated p53 inactivation was found to exist in HBV-related HCC tissues, and is positively correlated with unfavorable outcomes of patients^59,60^. Moreover, it was reported that HBx protein-mediated p53 inactivation was found throughout the whole process of the natural history of HBV infection, which can be detected in chronic hepatitis B (CHB), cirrhosis, and HCC, and might contribute to disease development. As Alfred S. L. Cheng et al.^61^ proposed, the overexpression of cyclooxygenase-2 (COX2) because of p53 inactivation is a media that accounted for disease development. COX2 can increase hepatic inflammation and promote collagen and TGF-β1 expression of hepatic stellate cells^62^, and also can prevent abnormal differentiation of HBV-infected hepatocyte apoptosis by COX2/Wnt/β‑catenin pathway^63^. In addition, HBx protein had been reported to aggravate liver inflammation of chronic hepatitis B by increasing MDM2 and decreasing p53^64^. Interestingly, HBV-infected hepatocytes can escape from immune-mediated injury but not from uninfected hepatocytes, because HBx protein can block tumor necrosis factor-α (TNF-α) and Fas-mediated apoptosis by activating NF-κB pathway^65^. Hence, HBx protein-mediated p53 inactivation might induce persistant liver inflammation that is not for HBV elimination. Certainly, p53 can mediate HBx protein inactivation through MDM2-dependent ubiquitin degradation and even seven in absentia homolog 1 (Siah-1) protein-dependent proteasomal degradation^66,67^. However, HBx protein has its defense mechanism by directly binding to MDM2 and promoting MDM2 translocation into the nucleus to antagonize p53 transcriptional activity^68^. In return, the stability of HBx protein can be strengthened by MDM2-mediated NEDDylation^69^. However, it is unfortunate that the combination of HBx protein and MDM2 might accelerate HBV-related fibrosis development. HBx protein can directly upregulate TGF-β1 expression via stabilization of the SMAD4 complex and activation of RAS–ERK and PI3K–AKT pathway^70^. Likewise, MDM2 can contribute to TGF-β1-induced fibroblast activation by attenuating Notch1 signaling^71^.

It is worth noting that HBx protein is not the unique protein of HBV, which can promote hepatoma cells’ transformation. Accumulating evidence suggests that HBeAg not only contributed to establishment of viral long-term infection, but also involved in HCC development. Indeed, chronic hepatitis B (CHB) patients with precore G1896A mutant, in which HBeAg production was blocked, have low risk of hepatocarcinogenesis^72^. Unlike the HBx protein, HBeAg is a nonstructural protein of HBV. The majority of HBeAg is secreted, although less than 30% of the mature HBeAg is retained in the cytoplasm^73^. The purposes of this precise strategy (secreting and retaining) are mainly contributing to HBV survival in hepatocytes. Secreted HBeAg can protect the injury of infected hepatocytes’ immune-system responses^72^, and the retained HBeAg can help infected hepatocytes escape from apoptosis by antagonizing the cytotoxicity from p53-dependent Fas/FasL and TRAIL^74^. Moreover, the carcinogenic effects of intracellular HBeAg were also found to be the consequence of compromised p53 activity. In the cytoplasm of infected hepatocytes, HBeAg can promote MDM2-mediated p53 ubiquitination and degradation, and impair the transcriptional activity of p53 by interacting with NUMB (a positive regulator of p53)^75^. Hence, HBV-infected hepatocytes cannot be killed because of retained HBeAg-mediated MDM2–p53 axis dysfunctions and secreted HBeAg-mediated immune-system exhaustion.

## HCV-induced hepatoma cells’ transformation

The full genetic information of HCV is localized in its positive-stranded RNA; unlike the HBV, the HCV genome does not need to enter the nucleus and integrate into the chromosome of infected hepatocytes^76^. Hence, HCV–RNA can be directly translated in the hepatocyte cytoplasm after endocytosis and uncoating, which are mediated by pH-dependent fusion with the lysosome^77^. Mechanistically, the HCV replication is mainly localized around the endoplasmic reticulum (ER). Mature HCV gene products (proteins) are mainly synthesized in the cytoplasm of hepatocytes, which can be distinguished into the structural and nonstructural proteins according to the spherical structure components of the virus^78^. The structural proteins were named capsid protein C, envelope glycoproteins E1 and E2, and protein P7; the nonstructural proteins were named NS2, NS3, NS4A, NS4B, NS5A, and NS5B^79^.

In recent years, multiple studies have proposed that HCV proteins are related with hepatocarcinogenesis, and those results were mainly from HCC tissues that have higher levels of HCV-related proteins than those in adjacent tissues^80^. Indeed, HCV has a strong ability to induce genomic instability and aneuploidy of hepatocytes, because the ways of its replication had affected the homeostasis of MDM2–p53 axis. As mentioned above, the transcription and new virus particle formation of HCV are processed around the ER, which could induce chronic ER and oxidative stress and NF-E2-related factor 2 (Nrf2) activation^81^. After activation, Nrf2, a nuclear factor, can induce MDM2-mediated Rb proteosomal degradation by promoting MDM2 expression, which might block the Rb/E2F pathway that mediated the inhibitor of cell-cycle progression of infected hepatocytes^82^. Hence, this procession could promote HCV long-term survival in infected hepatocytes with chronic ER and oxidative stress. Moreover, oxidative stress can further impair p53 activity by suppressing its acetylation and increasing MDM2-mediated ubiquitin degradation. Certainly, the liver has its defense mechanisms against persistent HCV infection. Kupffer cells and hepatic stellate cells (HSCs) can be activated by HCV-related proteins^78–83^. These cells can provoke the release of ROS of infected hepatocytes by producing pro-inflammatory (IL-1β, IL-6, and TNF-α) and profibrotic cytokines (TGF-β), which contribute to apoptotic cell death of infected hepatocytes via p53-dependent manner^84^. It is well recognized that the mechanisms of ROS induce apoptotic cell death via the upregulation of p53 function by increasing ubiquitin-dependent MDM2 degradation and thereby reducing p53 degradation^85^. However, HCV can overcome the ROS/p53-mediated apoptosis of infected hepatocytes by promoting promoter hypermethylation of p14, which can promote MDM2 accumulation, and inducing ub-mediated proteasomal degradation of p53^86^. In addition, in the absence of p53, HSCs might stimulate the polarization of Kupffer cells into a tumor-promoting state and enhance the proliferation of premalignant cells^87^. But more than that, HCV-induced p53 dysfunction has also been found in T cells that naive CD4^+^T-cell exhaustion can be induced by HCV via p53-dependent manner, which avoided infected hepatocytes to be killed by T cells. As Lam Nhat Nguyen et al.^88^ found, senescence and apoptosis are the main reasons of naive CD4^+^T-cell exhaustion because the stability and function of telomeric repeat-binding factor 2 (TRF2) was impaired by HCV-mediated upregulation of p53 expression; TRF2 is a key factor that can maintain telomere integrity and oppose replicate DNA damage, and TRF2 dysfunction would provoke cell senescence^89^. Hence, the above-mentioned evidence seems proposed that the normal proliferation and apoptosis of infected hepatocytes can be disrupted via HCV-induced apoptosis-related protein inactivation by interfering with MDM2–p53 axis; moreover, the abnormal differentiation hepatocytes cannot be killed because of T-cell exhaustion by HCV-mediated p53 dysfunction.

Apart from the above-mentioned mechanisms, the dysfunctions of anti- and pro-tumor proteins have been found to involve in HCV-induced HCC because of MDM2–p53 axis disruption. As Mirko Tarocchi et al.^90^ proposed, low expression of Krüppel-like C2H2 zinc finger (KLF) 6 may be a factor for hepatocarcinogenesis during chronic HCV infection, which was inhibited after persistant HCV replication. In general, KLF6 is classified into antitumor protein because it can suppress MDM2 transcription and promote p53 activation by interacting with P2 promoter of MDM2^91^. Hence, KLF6 dysfunction might be required for the abnormal proliferation of hepatocytes and contributes to hepatocarcinogenesis. Indeed, KLF6 dysfunction was associated with HCV-related HCC prognosis, and KLF6 was found to be abnormally decreased in HCC samples with a history of CHC^92^. Conversely, FUSE-binding protein 1 (FBP1) and protein kinase R (PKR) were found to be abnormally increased in HCC samples, both of which are associated with HCV replication and poor prognosis^93^. HCV can enhance FBP1 function and activate PKR, and thereby influence p53 expression and its DNA-binding activity. Hence, the function of p53 might gradually decrease along with HCV-related disease progression, which may be accounted for hepatoma cells’ transformation. However, p53 inactivation could disturb the glycometabolism of hepatocytes because of insulin resistance (IR), which can be provoked via HCV core protein by inhibiting PTEN/p53 pathway^94^. As a result, the energy supply of hepatocytes would switch from mitochondrial oxidative phosphorylation to Warburg glycolysis, which is the main metabolic way of hepatoma cells. Moreover, IR can induce steatohepatitis and liver fibrosis by promoting infected hepatocytes to produce inflammatory cytokines^95^.

## HIV-induced hepatoma cells’ transformation

Similarly to HCV, the full genetic information of HIV is localized in its single-stranded RNA^96^. Unlike with hepatitis viruses, HIV cannot survive in hepatocytes. However, patients with HIV infection are more prone to suffer from HCC than the general population^97^, and the HCC patients with HIV/hepatitis virus co-infection have higher HCC-staging score and a poor prognosis^98^.

Although multiple studies have confirmed that higher HIV RNA and long-term detectable HIV were independently associated with higher risk of HCC, especially in HIV patients with HBV/HCV infection^99^, yet the mechanism by which HIV induces HCC initiation has not been defined. Because the HIV is mainly infected with CD4^+^T cells and induced their depletion, researchers are focusing on the relationship between CD4^+^T-cell death and HCC initiation. Woo Park et al.^100^ proposed that the Nef (a protein of HIV) can be transferred to hepatocytes through conduits from infected CD4^+^T cells, and consequently contributed to dramatically augment ROS production and enhance ethanol- mediated HCV replication so as to accelerate HCC. In general, intrahepatic CD4^+^T cells have a strong power to control hepatocarcinogenesis by removing the senescent hepatocytes, and the loss of intrahepatic CD4^+^T cells can lead to abnormal hepatocyte growth^101,102^. However, to promote its survival in CD4^+^T cells, HIV has lots of ways against its normal defense mechanisms. Undoubtedly, HIV-mediated MDM2–p53 axis dysfunction was shown to be involved in CD4^+^T-cell exhaustion^103^. During acute HIV infection, the numbers of CD4^+^T cells are rapidly diminishing because of viral invasion and integration; after viral invasion and integration, DNA-dependent protein kinase (DNA-PK) can be activated and thereby causes phosphorylation of p53 and histone H2AX, both of which contributed to CD4^+^T-cell death^104^. However, the p53 pathway activation can exert a negative role in HIV replication^105^. Hence, HIV can degrade p53 and stabilize the post-translational level of MDM2 by inducing AKT-mediated MDM2 phosphorylation so as to maintain long-term survival time in CD4^+^T cells^106^. Apart from the CD4^+^T cells, Kupffer cells can also be infected by HIV. As a defense mechanism, macrophages can inhibit HIV replication in its cytoplasm by activating p53/p21/CDK1 pathway-mediated cell-cycle arrest^107^. However, HIV can negatively regulate the level of p53 by promoting the expression level of MDM2, which creates a cellular environment more favorable to the early steps of HIV-1 replication^108^. In addition, type I alpha-/beta-interferon (IFN-α/β) production of liver macrophages can be inhibited because HIV mediated p53/p21/IFN-stimulated gene (ISG) pathway inactivation^109^. Hence, Kupffer cells might present a low-affinity phenotype for eliminating the abnormal proliferation of hepatocytes. Taken together, although it does not have direct mechanisms that can explain HIV-induced HCC initiation and progression as the main hepatic supervisors of hepatocyte destructive–regenerative process, CD4^+^T-cell and Kupffer cell exhaustion might contribute to abnormal hepatocyte proliferation, especially in hepatocytes with hepatitis virus infections.

## MDM2–p53 axis dysfunction involved in metabolic syndrome-related HCC initiation

**Fig. 2:**
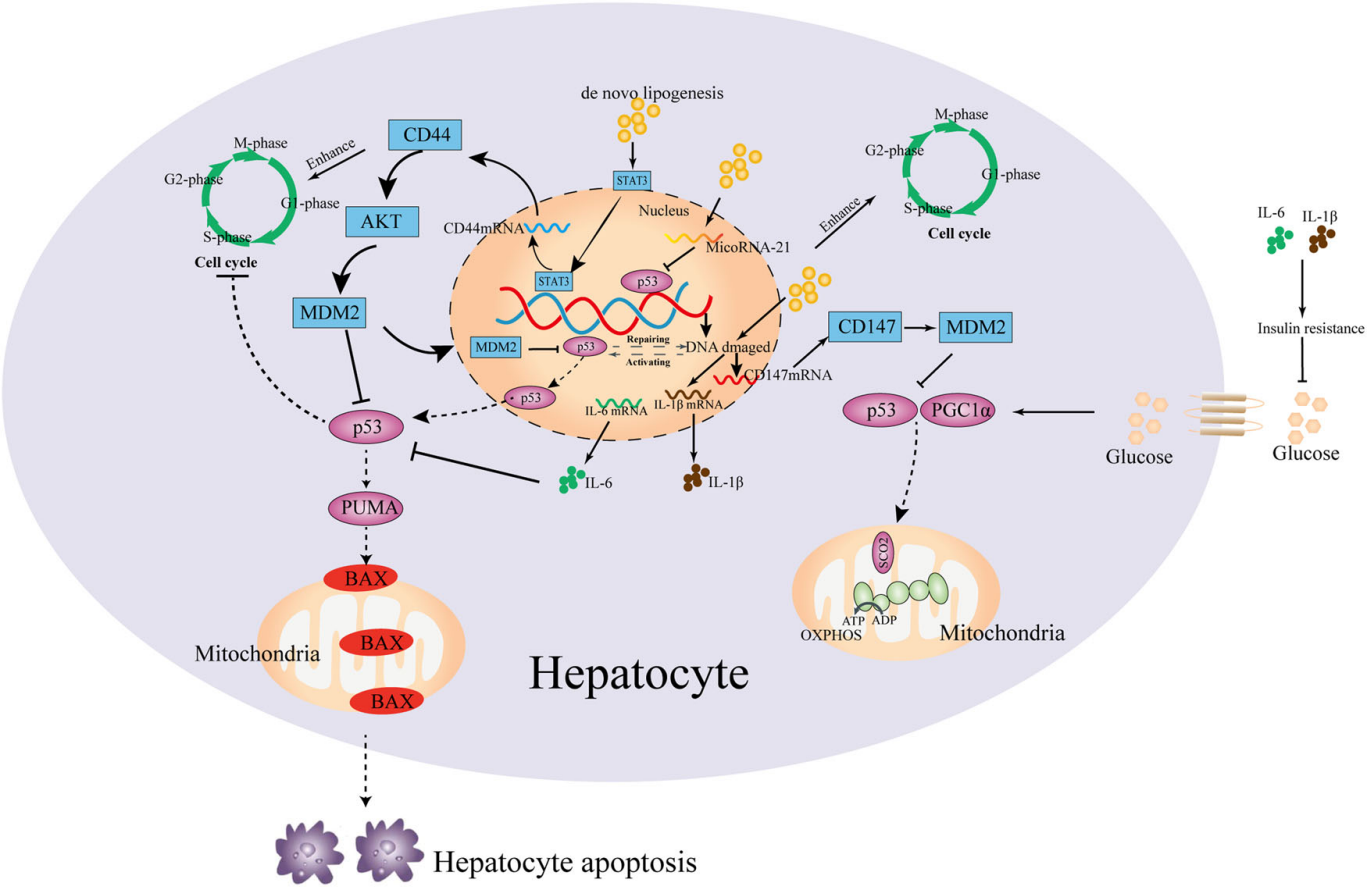
Glucose and lipid metabolism disorder disturbed normal hepatocyte program by inducing MDM2–p53 axis. After glucose and lipid metabolism disorder, the expression of CD44 is induced in hepatocytes with lipid deposition. Once expressed, CD44 potentiates AKT activation to induce the phosphorylation and nuclear translocation of MDM2, which terminates the p53 genomic surveillance response by repairing DNA or mediating cell apoptosis. Moreover, CD44 can directly prevent the premature cell-cycle exit and death of pericentral hepatocytes. Furthermore, lipid deposition can provoke DNA damage by lipotoxicity, which could contribute to the expression of pro-inflammatory cytokines (IL-6 and IL-1β) and CD147. CD147 could disturb mitochondrial biogenesis and oxidative phosphorylation in p53/PGC1a-dependent manner by promoting MDM2 expression. Pro-inflammatory cytokines can induce IR that aggravates glycometabolic disorder, and IL-6 can inhibit p53-mediated apoptosis. In addition, lipid deposition can increase the expression of microRNA-21, which can interfere with p53 binding to its DNA consensus sequence and enhance the cell cycle.

Caloric excess and sedentary lifestyle have led to metabolic syndrome^110^. Nonalcoholic fatty liver disease (NAFLD) and type 2 diabetes mellitus (T2DM) are strongly associated with metabolic syndrome, both of which are the independent risk factors for HCC development^111^. Similar to hepatitis virus infections, HCC can primarily arise in NAFLD patients without underlying cirrhosis, but the incidences of HCC are much higher^112^. Mechanistically, NAFLD and T2DM are mainly caused from fat accumulation and insulin resistance (IR), which are characterized by glucolipid metabolism disorder^113^. Considering that hepatocytes are accounted for glucolipid metabolism, the normal hepatocyte program could be disturbed by glucose and lipid metabolism disorder (Fig. 2). As discussed above, MDM2–p53 axis has involved in glucolipid metabolism, and its dysregulation might promote the metabolic disease-related liver disease development. Indeed, the functions of p53 protein were found to be abnormally changed in patients with NAFLD^114^. Mice with TP53 mutation can develop the spontaneous liver inflammation, steatosis, and fibrosis, also found in HCC patients with a history of NAFLD^115,116^. However, it seems that there is an absence of direct evidence that MDM2–p53 axis dysfunction promotes NAFLD to HCC. Recently, Heng Wu et al.^117^ proposed that microRNA-21 might be a potential link between NAFLD and HCC; they considered that microRNA-21 overexpression, which was mediated by lipid accumulation of hepatocytes, can enhance carcinogenesis-related protein expression (CCNB1, CCND1, and SREBP1C) by inhibiting p53 expression, because the target of microRNA-21 (HBP1) is also a transcriptional activator of p53. Moreover, the microRNA-21 overexpression can enhance G1/S and G2/S transition of hepatocytes with de novo lipogenesis by modulating Hbp1–p53 axis, and mircoRNA-21 knockdown prevents G1/S transition and cancer cell proliferation. In NASH-driven HCC models, tumors mainly arise from differentiated pericentral hepatocytes^118^, which exhibit higher levels of de novo lipogenesis than periportal hepatocytes^119^. Mechanically, early in tumor development, differentiated hepatocytes are converted into HCC progenitor cells (HcPC), in which its transcriptomic signature is similar to bipotential hepatobiliary cells that reside periportally^120^. However, there is lack of the mechanisms by which differentiated hepatocytes converted into HcPC. We discussed previously that inhibition of p53 accumulation and activity is a key early step in HCC initiation, which takes place long before p53-inactivating mutations are acquired^121^. Debanjan Dhar et al.^122^ proposed that the link of HCC initiation (differentiated hepatocytes converted into HcPC) and MDM2–p53 axis dysfunction is provided by CD44, which is a hyaluronic acid receptor whose expression is rapidly induced in carcinogen-exposed hepatocytes in a STAT3-dependent manner. Hepatic CD44 was strongly upregulated in NASH and HCC patients, and was expressed in HcPC but not in normal hepatocytes^123^. Once expressed, CD44 potentiates AKT activation to induce the phosphorylation and nuclear translocation of MDM2, which terminates the p53 genomic surveillance response^124^. Hence, CD44 will prevent the premature cell-cycle exit and death of pericentral hepatocytes that had acquired a potential oncogenic mutation in at least one DNA strand, and allows them to proliferate, duplicate the mutation, and transmit it into one of their progeny^121^. As a result, hepatocytes with de novo lipogenesis are prone to convert into HcPC, and the liver contains HcPC that undergoes multiple divisions to give rise to fully malignant HCC after long-term lipometabolic disturbance challenge^125^. In addition to serving as a HCC marker, CD44 may be involved in NASH development by inducing inflammation^123^. However, lipometabolic disturbance-induced liver inflammation could alter glucose metabolism of hepatocytes. Along with NASH progression, the expression of PGC1a can be gradually inhibited because of hepatic inflammation^126^. Under normal conditions, PGC1a can bind to p53 and enhances the p53-mediated cytochrome c oxidase assembly protein 2 (SCO2), which increases glucose utilization by increasing mitochondrial biogenesis and OXPHOS^127^. Downregulated PGC1α expression in hepatocytes could alter glucose metabolism, which might be related to hepatic dedifferentiation in HCC. Indeed, in human HCC lines, mitochondrial biogenesis and oxidative phosphorylation in p53/PGC1a-dependent way were downregulated because CD147 facilitated the cell surface expression of MCT1 and lactate export, which led to the activation of PI3K/AKT/MDM2 pathway and thus increased p53 degradation^128,129^. Interestingly, AKT–MDM2 pathway has another way to induce p53 inactivation: cytoplasmic sequestration, which was to be found in tissues from HCC patients with ASH and NASH. During the ongoing disease process, p53 inactivation by cytoplasmic sequestration was induced by LKB1 (serine/threonine protein kinase 11) that was related with HCC initiation^130^.

However, it is worth noting that MDM2–p53 axis maybe a double-edged sword in the progression of NAFLD^131^. In the early stages of NAFLD (liver steatosis), gradually increasing p53 response is a negative regulator of hepatic lipid accumulation^132^. But higher expression of p53 may induce liver inflammation, which would contribute to the progression of liver steatosis to more advanced stages (HCC) of NAFLD^133^. It was further confirmed that the expression of p53 in patients with liver steatosis without inflammation was significantly lower than that in NASH. However, the mechanism of MDM2–p53 axis that plays a negative role in progression of NAFLD is still unclear. In a recent study, Rui E. Castro et al.^134^ proposed that microRNA-34a overexpression is one of the reasons for liver steatosis without inflammation toward steatohepatitis or HCC; microRNA-34a overexpression will increase p53 acetylation and transcription, which leads to the diminishment of sirtuin 1 (SIRT1) in the NAFLD liver. However, as a NAD-dependent deacetylase that modulates apoptosis of hepatocytes in response to oxidative and genotoxic stress, SIRT1 inactivation could provoke insulin resistance and pro-inflammatory cytokine (TNF-α, IL-1β, and IL-6) expression and pro-inflammatory signaling pathway (STAT3 and JNK) activation^135^, all of which are important factors governing hepatoma cells’ transformation. It is well-known that pro-inflammatory cytokines that induced chronic inflammatory response have to be considered as two potent activators of pro-oncogenic signaling that were linked to HCC onset^136^, such as IL-6 prevents DNA-damage-induced hepatocyte apoptosis through suppression of p53 and enhances β-catenin activation^137^. Hence, it seems reasonable that UDCA was found to be associated with suppressor of HCC, because UDCA can ameliorate insulin sensitivity and liver inflammation by stabilizing the p53/MDM2 interaction and enhancing MDM2-dependent ubiquitination of p53^138^.

## Therapeutic avenues that target MDM2–p53 axis in HCC

As discussed above, the dysfunction of MDM2–p53 axis plays a critical role in HCC tumor development and progression. Hence, normalization of MDM2–p53 axis has been considered as an exciting target for antitumor drug design. The main points of therapies are focusing on restoring p53 function, decreasing MDM2 function, and normalizing MDM2–p53 protein–protein interaction^139^. In addition, considering that the p53 mutation also accounted for HCC development, some therapeutic avenues can also exert the role of antitumor by antagonizing MDM2 independent of p53.

## Anti-HCC by restoring p53 function and normalizing MDM2–p53 interaction

Until now, there are some strategies that have been considered in p53 reactivation^140^, including disrupting the MDM2–p53 interaction, releasing p53 by small molecules, restoring wild-type function to mutant p53 by covalent modification of its core domain, and reconstructing a functional copy of p53 by viral or nonviral DNA transfection. In clinical application, recombinant adenovirus—p53 is a relatively safe and effective method for treating HCC^141^, and sequential therapy of p53 gene transcatheter arterial infusion was safe and could prolong the survival time of the patients^142^. However, it should be noted that it is lack of MDM2–p53 axis-targeted drugs that can be used in patients with HCC. As the standard first-line systemic therapy for HCC, the antineoplastic functions of sorafenib are partly associated with upregulation of p53^143^. In recent studies, some antineoplastic agents that can activate p53 pathway have been found in vitro. Cell-cycle arrest and apoptosis of HCC1419 (mutp53–Y220C) can be provoked by SLMP53-2, which is a new molecule to activate mutation of p53 by enhancing its interaction with the heat-shock protein 70 (Hsp70) and leading to the reestablishment of p53 DNA-binding ability as well as wild-type p53^144,145^. In another p53-null HCC line, the cell growth of Hep3B cells can be inhibited by melatonin (a hormone for regulation of circadian rhythms), which can promote the expression of several proapoptotic target genes (p21 and BAX) of p53^146^. Likewise, it might exert antineoplastic effects by improving the wide-type p53 function in HCC. HepG2 cells can be killed by indolizine derivatives, which promoted p53 activation and increased its accumulation in nuclei^147^. As a new wild-type p53 activator, adiponectin can significantly attenuate HCC progression of rats by targeting p53/TRAIL/caspase-8 signaling^148^. However, it is worth noting that WTp53 activation might have a counterintuitive effect to enhance tumorigenesis by promoting cancer metabolic switch via p53/PUMA-dependent oxidative phosphorylation suppression^149^. Moreover, elevated p53 could lead to decrease the tumor-protective function of the estrogen alpha (ERα) pathway in female hepatocarcinogenesis^150^, although ERα pathway was found to be involved in the development of chronic liver diseases^151^. Hence, some antineoplastic drugs/targets have been constructed/considered to antagonize MDM2, independent of p53. MDM2 and nuclear factor of activated T-cell 1 (NFAT1) dual inhibitor induces MDM2 autoubiquitination and degradation, and represses NFAT1-mediated MDM2 transcription, which can inhibit the growth and metastasis of HCC cells in vitro and in vivo^152^. SP141 and MA242 (MDM2 inhibitors) are the potential drugs for treating HCC, because their effectiveness had been confirmed in breast cancer models and pancreatic tumor mice^153,154^.

However, considering the side effects and resistance of antineoplastic drugs of HCC treatment, some new extracts from common plants can directly antagonize HCC growth, improve the anticancer activity, and reduce hepatotoxicity by enhancing p53 function and disrupting MDM2–p53 interaction. Oleanolic acid can help cisplatin to overcome the chemotherapeutic resistance by activating p53/Bax/cytochrome C/caspase-3 proapoptotic signaling pathway^155^. Acetylshikonin can directly promote HepG2 cell apoptosis by activating p53/PUMA/Bax pathway^156^. Troxerutin can subdue hepatic tumorigenesis via disrupting the MDM2–p53 interaction by decreasing the expression of MDM2 and increasing that of wild-type p53^157^.

## Potential therapeutic targets of anti-HCC

There are lots of potential targets that were related with MDM2–p53 axis dysfunction that can be considered to exert anti-HCC properties, all of which have been found to be abnormally changed in tissues of HCC patients, and are correlated with favorable/unfavorable HCC outcomes (Table 1). Among those favorable prognosis-related molecules, the anti-HCC activity of RAD52 motif 1 (RDM1), adipose triglyceride lipase (ATGL) and growth arrest-specific protein 2 (GAS2), and microRNA-34a and microRNA-621, was mainly associated with increased p53 function (transcription and activating p53 pathway). RDM1 promotes the expression of p53 downstream targets (p21, cyclinA1, and 14-3-3δ) and inhibits activation of Ras/Raf/ERK signaling pathway depending on p53^158^. ATGL imposes glycolytic rewiring of HCC by promoting acetylation and stabilization of p53 throughout ATGL/PPAR-α/p300 axis^159^. GAS2 induces SK-Hep1 apoptosis via p53-dependent apoptosis pathway. MiR-34a enhances the inhibitory effect of doxorubicin on HepG2 cells by promoting p53-mediated multidrug-resistance protein (MDR)1/P glycoprotein (P-gp) inhibitor^31^. MicroRNA-621 improves the radiosensitivity of HCC cells by directly targeting SET domain-bifurcated 1 (SETDB1) and thereby activating the p53 pathway^160^. In addition, RNA-binding motif protein 38 (RBM38), glycogen synthase 2 (GYS2), and sirtuin3 have been reported to exert anti-HCC roles via stabilizing the MDM2–p53 loop function by inhibiting MDM2 and restoring wtp53 expression and/or slowing p53 degradation^58,161,162^.

**Table 1:** Overview of potential therapeutic targets of anti-HCC by modifying MDM2–p53 axis.

Targets	Mechanism	Effects of HCC	Refs.
Lgr5, PRC1, SIRT7, and MYL6B	Inducing p53 degradation via MDM2, disrupting its stabilization, and inhibiting its translocation	Increasing cell migration and resistance to doxorubicin, inducing EMT, and promoting cytokinesis in HCC cells	^53,163–165^
MiR-34a, MiR-621, RBM38, GYS2, Sirtuin3, RDM1, ATGL, and GAS2	Enhancing the transcriptional activity of p53 and activating its pathway	Inhibiting activation of Ras/Raf/ERK signaling pathway, impeding energy supply of HCC from glycolysis, and sensitizing HCC to doxorubicin and radiosensitivity	^32,59,158–161^

*LGR-5* leucine-rich repeat-containing G-protein-coupled receptor 5, *PRC1* protein regulator of cytokinesis 1, *SIRT7* sirtuin 7, *MYL6B* protein myosin light chain 6B, *RBM38* RNA-binding motif protein 38, *GYS2* glycogen synthase 2, *RDM1* RAD52 motif 1, *ATGL* adipose triglyceride lipase, *GAS2* growth arrest-specific protein 2.

Conversely, as unfavorable factors for HCC prognosis, the upregulated expression of molecules can directly promote hepatoma cell growth and indirectly facilitate HCC development by inducing chemotherapy resistance, all of which are mainly associated with decreased p53 activation. Protein regulator of cytokinesis 1 (PRC1) can promote cytokinesis of HCC cells and induce HCC cell desensitization to taxol by inhibiting p53/p21 or p53/p14ARF activation^163^; SIRT7 can cause doxorubicin resistance by interacting and inducing deacetylation of p53, and thereby reducing affinity for the NOXA promoter and its transcription^164^. In addition, protein myosin light chain 6B (MYL6B) can promote HCC development by binding MDM2 and p53 proteins, accelerating the p53 degradation^165^.

## Conclusion

This review summarized the current literature highlighting the negative effects of MDM2–p53 axis dysfunction in the context of hepatoma cells’ transformation (Table 2). Although the high number of studies had proposed some tumor cell-specific signaling pathways that modulate the MDM2–p53 axis, the mechanisms of the MDM2–p53 axis dysfunction in HCC progression are complex. In addition, future studies should focus on the hallmarks of MDM2–p53 axis dysfunction in the chronic progressive liver disease that is strongly associated with HCC initiation and development, which will help to diagnose and treat the HCC at vulnerable early stages. At the same time, we should focus on the expression levels of some molecules that can modulate the balance of MDM2–p53 axis in chronic liver diseases, which had been found to be abnormally changed in HCC tissues. By focusing on the MDM2–p53 axis dysfunction in the transformation of normal hepatocytes into hepatoma cells, we hoped to provide some clues for the liver cancer prevention and pre-HCC treatment strategies.

**Table 2:** The changes in antitumor and carcinogenic factors following MDM2–p53 axis dysfunction.

	Viruses induced			
Status	HBV^refs^	HCV^refs^	HIV^refs^	Metabolic syndrome-induced
Promoting HCC (increased)	NTCP^42^, LGR-5^53^, SNORA18L5^54^, COX2^61^, TGF-β1^70,71^, and NUMB^75^	ER stress and OS^81^, Nrf2^82^, FBP1^93^, and IR^95^	ROS^100^	MicroRNA-21^117^, CD44^120–123^, CD147^128,129^, LKB1^130^, and IL-6^137^
Inhibiting HCC (decreased)	MicroRNA-148a^57^, Fas/FasL, and TRAIL^74^	Rb^82^, Kupffer^87^, CD4^+^T^88^, and KLF6^90^	CD4^+^T^104^ and Kupffer^108,109^	PGC1a^127^ and SIRT1^134^

*NTCP* sodium taurocholate cotransporting polypeptide, *LGR-5* leucine-rich repeat-containing G-protein-coupled receptor 5, *COX2*, cyclooxygenase-2, *Nrf2* NF-E2-related factor 2, *ER stress* endoplasmic reticulum stress, *OS* oxidative stress, *IR* insulin resistance, *FBP1* FUSE-binding protein 1, *Rb* ribosome, *TRF2* telomeric repeat-binding factor 2, *KLF6* Krüppel-like C2H2 zinc finger 6, *ROS* reactive oxygen species, *LKB1* serine/threonine protein kinase 11, *SIRT1* sirtuin 1.
